# International conference on Cell Death in Cancer and Toxicology 2018 (CDCT-2018)

**DOI:** 10.1186/s12964-018-0238-x

**Published:** 2018-06-28

**Authors:** Kausar M. Ansari, Praveen R. Arany, Eli Arama, Sarit Larisch, Raymond B. Birge, Dhyan Chandra

**Affiliations:** 1 Food, Drug, and Chemical Toxicology Group, CSIR-Indian Institute of Toxicology Research, Vishvigyan Bhawan, 31, Mahatma Gandhi Marg, Lucknow, Uttar Pradesh 226001 India; 2 The University at Buffalo, New York, USA; 3 Department of Molecular Genetics, Weizmann Institute of Science, Rehovot, 76100 Israel; 4 University of Haifa, Haifa, Israel; 5 Rutgers University, Cancer Institute of New Jersey, Newark, NJ, USA; 6 Department of Pharmacology & Therapeutics, Roswell Park Comprehensive Cancer Center, Elm & Carlton Streets, Buffalo, NY 14263 USA

**Keywords:** Cell death, Cancer, Toxicology, Cancer stem cells, Mitochondria, Therapy resistance, Phytochemicals, Apoptosis, Immune therapy, Metastasis, and Genomics

## Abstract

The International Conference on Cell Death in Cancer and Toxicology 2018 (February 20–22, 2018) provided an international forum for scientific collaborations across multiple disciplines in cancer, cell death, and toxicology. During the three-day symposium, researchers and clinicians shared recent advances in basic, clinical, and translational research in cancer. Several student poster abstracts were selected for platform talks and many young investigators participated in the meeting. Together, this highly interactive meeting showcased the rapid expansion in biomedical research in India and paved the way for future meetings on cell death and cancer throughout India.

The International Conference on Cell Death in Cancer and Toxicology (CDCT) (http://www.cdct2018.com) was held in Lucknow, India, from February 20–22, 2018. The meeting was organized by Dr. Kausar M. Ansari (CSIR-Indian Institute of Toxicology Research (IITR), Lucknow, India) and Dr. Dhyan Chandra (Roswell Park Comprehensive Cancer Center, Buffalo, USA) as part of a joint collaboration with the International Cell Death Society, USA (https://celldeath-apoptosis.org). The 3-day symposium fostered international collaborations in the fields of cell death, cancer, and toxicology, as well as showcased major advancements in biomedical sciences in India over the past decade. The meeting structure provided a forum for students and junior investigators in India to interact with leaders of their fields, advancing scientific research and developing future collaborations. Over 200 attendees participated in the symposium including researchers, students, advocates and media, and over 75 invited speakers presented recent advances in basic, clinical, and translational and research related to cell death and cancer. CDCT-2018 also included over 80 poster presentations that encouraged one-on-one discussion, informal interactions between researchers, and new collaborations. Several poster abstracts were selected for platform talks, and for the young investigators, outstanding oral and poster presenters were awarded during the closing ceremony. The proceeding of the CDCT 2018 was published in the Cancer Medicine (Volume 7, Issue S1, 19 February 2018) and was released during the meeting.

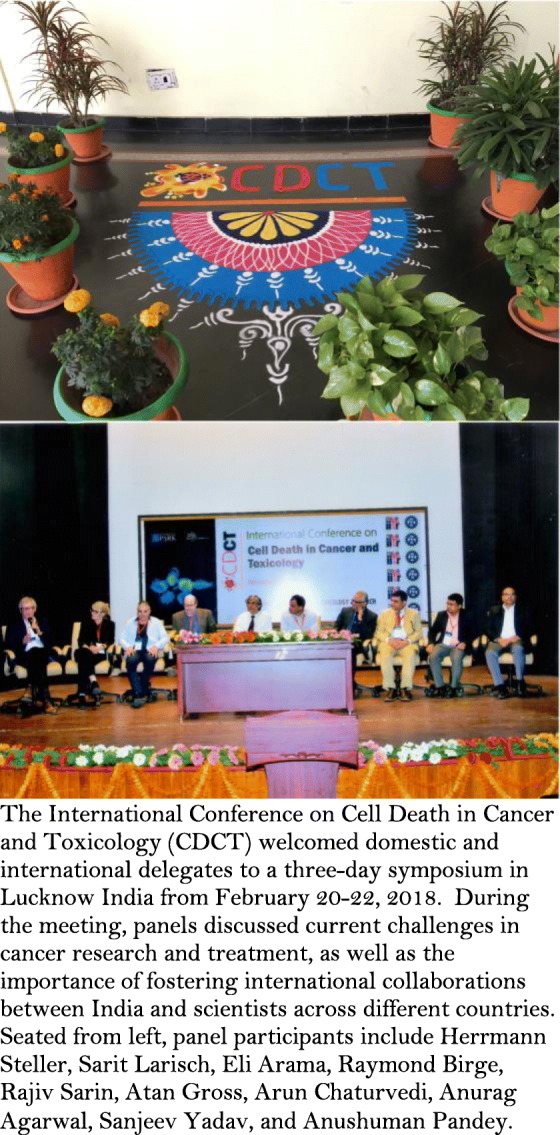



The conference began with lamp lighting tradition and honoring several delegates and invited speakers. The inauguration session was followed by a keynote talk from Dr. Hermann Steller (The Rockefeller University, USA) and three plenary sessions. Dr. Steller discussed how apoptosis-induced proliferation (AIP) and apoptosis induced apoptosis (AIA) are coordinated during developmental processes, and discussed how new non-canonical pathways from dying cells are communicated to neighboring cells. Dr. Eli Arama (Weizmann Institute of Science, Israel) presented his recent work unraveling a caspase-independent, alternative cell death pathway highlighting the importance of a lysosomal nuclease in triggering DNA damage response and the consequent developmental cell death, which may have an unanticipated impact in targeting cancer cells. Finally, Dr. Sarit Larisch (University of Haifa, Israel) described her recent work on the pro-apoptotic ARTS protein, which induces apoptosis through degradation of the two major prosurvival proteins XIAP and Bcl-2, and how ARTS mimetics, with similar functions can kill cancer cells.

The second plenary session was initiated by a keynote talk from Dr. Atan Gross (Weizmann Institute of Science, Israel) who described the role of mitochondrial dynamics in homeostasis and cell death and focused on the functional significance of mitochondrial protein MTCH2 in lipid homeostasis. Dr. Dhyan Chandra (Roswell Park Comprehensive Cancer Center, USA) continued on the theme of mitochondria and discussed recent updates on the regulation of cell death by oxidative phosphorylation (OXPHOS) and whether combination therapy can be formulated based on OXPHOS-targeting anticancer agents. Similarly, Dr. Rana P. Singh (Jawaharlal Nehru University, India) highlighted the importance of nontoxic anticancer agent fisetin targeting OXPHOS Complex I, producing reactive oxygen species and inducing cell death in cancer. Other mitochondrial pathways involving mitochondrial pore machinery were discussed, which particularly have implications in overcoming resistance to current anticancer therapeutics.

In another plenary keynote lecture, Dr. Raymond Birge (Rutgers University, Cancer Institute of New Jersey) continued on a theme developed by Dr. Steller that apoptotic cells are not functionally inert in tissues, but have important signaling and immunological consequences as a result of apoptotic cell clearance (efferocytosis). Dr. Birge emphasized that targeting phosphatidylserine and phosphatidylserine receptors (TAM Receptor) may act akin to immune checkpoint inhibitors to stimulate anti-tumor host immunity. Dr. Levi Beverly (James Graham Brown Cancer Center, USA) highlighted the importance of metastasis suppression in human lung adenocarcinoma. The issue of metastasis was a recurrent theme during the meeting, since the vast majority of patients succumb to cancer from metastatic disease, calling for more research into the genetics and novel treatments such as immunotherapy.

The first day of the symposium concluded with a very active panel discussion on current and future perspectives in cancer therapeutics. The panel discussion was led by Dhyan Chandra, where panelists (Hermann Steller, Sarit Larisch, Eli Arama, Raymond Birge, Rajiv Sarin, Atan Gross, Arun Chaturvedi, Anurag Agarwal, Sanjeev Yadav, and Anshuman Pandey, Manoj Kumar, Eli Arama and Anand Narain Srivastava) discussed the current cancer treatment challenges in India and across the world. Several important themes were discussed including early detection, immune therapy, combination therapy, personalized therapy, and how basic knowledge of cell death mechanisms can be exploited for developing better therapies for cancer. Panelists agreed that there is an urgent need to foster long-term collaboration between clinical and basic researchers so that newer treatment strategies can be formulated for future personalized medicine for cancer cure and management.

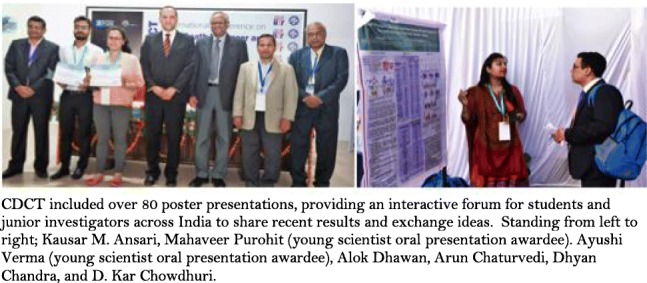



Many speakers including Drs. Nagaraj Balasubramaniam, Mayurika Lahiri, Amit Kumar, Manoj K Pandey, Dinesh Kumar Singh discussed on various aspects of metastasis, angiogenesis, tumor microenvironment, reactive oxygen species, and DNA damage response in cancer cells. For example, how adhesion dependent trafficking pathway, microenvironment, transcriptional regulatory pathway, mTOR signaling pathway, DNA-dependent proteins kinases could be targeted for disruption of cancer growth, survival, proliferation, invasion, and migration in cancer. Therapy resistance and cancer recurrence is one of the main reasons for cancer-related death among humans. Accordingly, various speakers discussed the importance of ubiquitination, microRNA, and cancer stem cells in cell death resistance leading to cancer recurrence. Using an enzyme-based high throughput screening strategy, Dr. Xinjiang Wang (Roswell Park Comprehensive Cancer Center, USA) identified different chemical classes of small molecule inhibitors for Mdm2-Mdm4 RING domain designated as MMRi, which showed potent pro-apoptotic activity in drug resistant leukemia/lymphoma cells. Dr. Srinivasa Murty Srinivasula (Indian Institute of Science Education and Research, Thiruvananthapuram, India), Dr. Kumar Somasundaram (Indian Institute of Science, Bangalore), and Dr. Bhudev Chandra Das (AMITY University, NOIDA, India) outlined the unique metabolic, signaling networks, and altered genetic and epigenetic networks within cancer stem cells. They emphasized these key cancer stem cells characteristics could be potentially targeted with novel treatment approaches modulating proteostasis and mRNA modifications, that not only target the primary tumor but also targets secondary and residual tumors to prevent relapses in cancer patients. In the follow up talks, Dr. Dipak Datta (CSIR- Central Drug Research Institute, India), Satyendra Kumar Singh (King George’s Medical University, India) and Hifzur Siddique (Aligarh Muslim University, India) spoke about the key roles of EZH2, Gfi-1 and Numb in cancer stem cell biology.

The overall discussion on other talks was tailored towards the functional interplay between micro-RNA and EZH2, which may devise newer strategies to control malignancies in human. Besides these conventional approaches, newer approaches using various biophotonics therapies have shown much promise in these areas. These low dose treatments are currently being used in conjunction with conventional oncotherapies to target tumor cells themselves or the supporting cells within the tumor niche, noting the effects on the vascular perfusion and host immune responses. A talk by Praveen Arany (University at Buffalo, USA) outlined cell death mechanisms mediated by ATF4 observed in phototoxic responses with near-infrared light. Various other targets and compounds were discussed that could sensitize cancer stem cells leading to prevention of cancer recurrence. Overall, presentations in these sessions highlighted the current understanding and potential values of targeting cancer stem cells and other signaling during tumorigenesis for the improvement of cancer therapy.

Since cancer cells develop resistance via evasion of apoptotic cell death, restoring cell death in cancer has always been the key objective of many scientists. Dr. Ruth Kluck (Walter and Eliza Hall Institute for Medical Research. Australia) described the underlying mechanism of controlling apoptotic cell death by regulating mitochondrial pore formation. The findings help characterize how proapoptotic Bak and Bax become sequestered by prosurvival proteins, and the role this sequestration might play in chemoresistance. The Warburg effect and metabolism are also important aspects of cancer cell survival, and Dr. Sanjeev Shukla (Indian Institute of Science Education and Research, Bhopal, India) elaborated novel mechanism for reversing Warburg effect and inhibiting breast cancer cell growth. Talks were also presented to establish how novel metabolic mechanism mediates chemotherapy resistance in pancreatic and other cancer types. Dr. Amit Dutt discussed how genomics data from Indian origin samples, along with discovery of novel molecular subclasses could be useful for new therapeutic targets and biomarkers for clinical development. Various toxicological responses and chemopreventive effect of some phytochemicals were discussed during the meeting specially the role of polymorphism in cellular toxicity, mechanisms of cytotoxicity upon exposure to toxic agents such as sulfur mustard, and how phytopharmaceuticals could help design better cancer therapy.

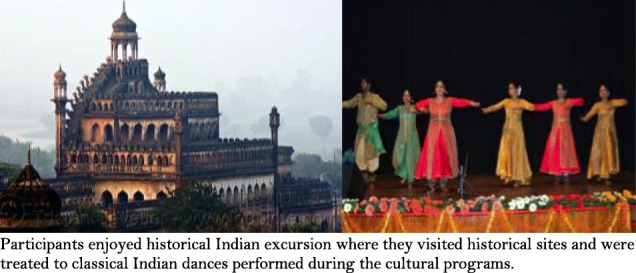



The meeting was concluded in a closing ceremony in which Drs. Alok Dhawan (CSIR-IITR), Kausar M. Ansari (CSIR-IITR), Arun Chaturvedi (King George’s Medical College), D Kar Chowdhuri (CSIR-IITR) and Dhyan Chandra (Roswell Park Comprehensive Cancer Center) discussed the importance of future meetings in India to establish collaborations between Indian scientists and scientists across the world. Funding institutions such as the Science & Technology International Cooperation Division of Department of Science & Technology (http://www.dst.gov.in/international-st-cooperation) that establish bilateral agreement between USA, France, and Germany for promoting workshops should be expanded with increased funding for project development, scientific innovation, and student fellowships. This highly interactive meeting opened new avenues for collaborations, paved the way for future meetings on Cell Death and Cancer in India, and provided a culturally enriching experience for all.

## Abbreviations

CDCT: Cell Death in Cancer and Toxicology
IITR: CSIR-Indian Institute of Toxicology Research

